# Contribution of the Ly49E Natural Killer Receptor in the Immune Response to *Plasmodium berghei* Infection and Control of Hepatic Parasite Development

**DOI:** 10.1371/journal.pone.0087463

**Published:** 2014-01-30

**Authors:** Jessica Filtjens, Lander Foquet, Sylvie Taveirne, Els Van Ammel, Mandy Vanhees, Aline Van Acker, Tessa Kerre, Tom Taghon, Bart Vandekerckhove, Jean Plum, Philippe E. Van den Steen, Georges Leclercq

**Affiliations:** 1 Department of Clinical Chemistry, Microbiology and Immunology, Ghent University, Ghent, Belgium; 2 Laboratory of Immunobiology, Rega Institute for Medical Research, University of Leuven, Leuven, Belgium; Agency for Science, Technology and Research - Singapore Immunology Network, Singapore

## Abstract

Natural killer (NK) cells have different roles in the host response against *Plasmodium*-induced malaria depending on the stage of infection. Liver NK cells have a protective role during the initial hepatic stage of infection by production of the T_H_1-type cytokines IFN-γ and TNF-α. In the subsequent erythrocytic stage of infection, NK cells also induce protection through Th1-type cytokines but, in addition, may also promote development of cerebral malaria via CXCR3-induction on CD8^+^ T cells resulting in migration of these cells to the brain. We have recently shown that the regulatory Ly49E NK receptor is expressed on liver NK cells in particular. The main objective of this study was therefore to examine the role of Ly49E expression in the immune response upon *Plasmodium berghei* ANKA infection, for which we compared wild type (WT) to Ly49E knockout (KO) mice. We show that the parasitemia was higher at the early stage, i.e. at days 6–7 of *Plasmodium berghei* ANKA infection in Ly49E KO mice, which correlated with lower induction of CD69, IFN-γ and TNF-α in DX5^−^ liver NK cells at day 5 post-infection. At later stages, these differences faded. There was also no difference in the kinetics and the percentage of cerebral malaria development and in lymphocyte CXCR3 expression in WT versus Ly49E KO mice. Collectively, we show that the immune response against *Plasmodium berghei* ANKA infection is not drastically affected in Ly49E KO mice. Although NK cells play a crucial role in *Plasmodium* infection and Ly49E is highly expressed on liver NK cells, the Ly49E NK receptor only has a temporarily role in the immune control of this parasite.

## Introduction

Malaria is a global disease that resulted in more than 650,000 deaths in 2010 (www.who.int). It is caused by protozoan *Plasmodium* parasites that are transmitted by infected *Anopheles* mosquitoes. The *Plasmodium* sporozoites enter the body and invade dermal blood vessels to reach the bloodstream, after which they migrate to the liver, initiating the hepatic stage of the infection. Sporozoites invade hepatocytes and develop into mature liver schizonts, each containing multiple merozoites. After schizont rupture, merozoites invade red blood cells (RBCs) leading to the symptomatic phase of infection, also called the erythrocytic stage. Here, a cyclic phase starts in which merozoites develop in the RBC as ring forms, followed by the progression to trophozoites and eventually to mature schizonts. Rupture of these schizonts and lysis of the RBCs results in release of a new wave of merozoites, which invade other RBCs [1]–[3].

Roland *et al*. [4] showed that hepatic and splenic natural killer (NK) cells are activated during the early phase of infection with *Plasmodium yoelii* sporozoites. This results in an increase in the number of liver NK cells and in production of the T_H_1-type cytokines IFN-γ and TNF-α by splenic and hepatic NK cells. Liver NK cells are cytotoxic against the *Plasmodium* liver stages, but not against the *Plasmodium* erythrocytic stages. These *in vitro* data indicating a protective role of NK cells were confirmed by *in vivo* experiments, showing that NK cells are particularly involved in the early immune response controlling the hepatic phase of *Plasmodium* infection [4]. In the erythrocytic phase, NK cells also contribute to the control of parasitemia by producing Th1 cytokines, which further potentiate e.g. phagocytosis by macrophages [5]; [6]. Finally, NK cells have been shown to promote development of murine cerebral malaria, as NK cell depletion results in inhibition of T cell recruitment to the brain of *Plasmodium berghei*-infected animals, which correlates with the down-regulation of the chemokine receptor CXCR3 [7].

NK cells display a broad repertoire of activating and inhibitory cell surface receptors, which regulate NK cell activity. In mice, inhibitory NK receptors, including members of the Ly49 receptor family and the CD94/NKG2A receptor, bind major histocompatibility complex (MHC) class-I and MHC class I-like molecules. Signaling by these receptors occurs via cytoplasmic immunoreceptor tyrosine-based inhibitory motifs. Activating receptors, on the contrary, possess a charged transmembrane residue and couple to the immunoreceptor tyrosine-based activating motif-bearing adaptor molecules DAP12, CD3ζ or FcεRγ [8]–[10]. NK cell activation occurs when MHC class-I molecules on transformed or infected cells are absent, eliminating the inhibitory signal (“missing-self recognition”), or when ligands for activating NK receptors are upregulated (“induced-self recognition”) [11]–[15].

Ly49E is a unique member of the murine Ly49 NK receptor family that displays characteristics that clearly distinguish this receptor from other Ly49 family members. Unlike other NK receptors, Ly49E fails to bind classical MHC class-I ligands [16]. Instead, we showed that this receptor is triggered by urokinase plasminogen activator (uPA), a non-MHC class-I molecule [17]. Also, Ly49E is the only Ly49 receptor expressed on fetal and neonatal NK cells [18]; [19]. In contrast, in adult tissues Ly49E expression is restricted to skin Vγ3 T cells [20]; [21] and intestinal intraepithelial T lymphocytes [22]; [23]. Ly49E is largely absent on conventional peripheral NK cells in adult mice [19]; [20]; [24]. However, DX5^-^ liver NK cells express high levels of Ly49E, and this both in fetal, newborn and adult mice [25].

As NK cells play a crucial role in the immune response controlling *Plasmodium* infection [4]–[6], and as we have recently shown that murine liver NK cells express high levels of the Ly49E receptor [25], we hypothesized that Ly49E plays a regulatory role in the NK response during this infection. As we have recently generated an Ly49E knockout (KO) mouse [25], we now have an ideal model to study this hypothesis. We here examined the immune response, parasitemia, cerebral malaria and survival of Ly49E KO vs wild type (WT) mice upon infection with *Plasmodium berghei* sporozoites.

## Materials and Methods

### Mice and *Plasmodium berghei* infection

WT and Ly49E KO mice (C57BL/6 background) were bred and housed in our SPF animal facility in individually ventilated cages with spruce chips bedding, with a maximum of 5 mice per cage. Male mice from different breeding pairs were never mixed to avoid fighting of the mice. Water and food was available ad libitum and was checked daily.

*Plasmodium berghei* ANKA 676m1cl1 (*P. berghei* ANKA), which expresses a fusion protein of green fluorescent protein (GFP) and firefly luciferase, was used to infect the mice. Sporozoites were obtained by dissecting the salivary glands of *P. berghei* ANKA-infected mosquitoes. The mosquitoes were kindly provided by Dr. Robert Sauerwein (Nijmegen, The Netherlands). An equal number of male and female mice at the age of 6 to 8 weeks were infected with sporozoites, and mice were age matched between WT and Ly49E KO mice. Mice were infected intravenously (i.v.) with 10,000 sporozoites. 0.375 mg/ml 4-aminobenzoic acid (PABA; Sigma-Aldrich, St. Louis, MO, USA) was added to the drinking water twice a week to ensure optimal parasite growth. All animal experimentation was performed after approval of the Animal Ethical Committee at the Faculty of Medicine and Health Sciences of Ghent University, Ghent, Belgium (ECD 12/07).

### Parasitemia, scoring of cerebral malaria and humane endpoints

Liver parasitemia was visualized through whole body imaging using the *in vivo* Imaging System (IVIS Lumina II; Perkin Elmer, USA). Animals were anesthetized using isoflurane anesthesia, the abdomen was shaved and D-luciferin dissolved in PBS (150 mg/kg body weight; Caliper Life Sciences, USA) was injected intraperitoneally in the abdomen. Animals were kept anesthetized during the measurements, which were performed within 10 minutes after the injection of D-luciferin. Bioluminescence imaging was acquired after an exposure time of 120 seconds. Luminescence results are expressed as relative levels of luminescence. Blood parasitemia was determined daily by flow cytometry. One drop of blood (approximately 3 µl) was collected from the tail vein in an EDTA-coated tube (Microvette® 500 K3E, Sarstedt, Essen, Belgium) filled with 47 µl PBS. Samples were diluted 10-fold in PBS and subsequently measured on a FACSCalibur (BD Biosciences, San Jose, CA, USA) to determine the percentage of GFP-positive, and thus *P. berghei* ANKA-infected, RBCs. Mice were judged to develop cerebral malaria if they displayed neurological signs such as loss of reflex, ataxia and paralysis. Mice developed cerebral malaria between days 7 and 11 post-infection (p.i.). In this period of time, animals were checked 3 times a day and the mice were sacrificed by cervical dislocation as soon as the indicated neurological signs were visible. Mice not affected by cerebral malaria were sacrificed by cervical dislocation when parasitemia reached 20–30%. We did not use analgesics because animals were checked often enough to intervene when animals were suffering.

### Cell preparation

Spleen and liver lymphocytes were isolated from non-infected and from *P. berghei* ANKA-infected WT and Ly49E KO mice that were sacrificed by cervical dislocation at days 5 and 7 p.i. Spleens were disrupted, minced and passed through a 40 µm cell strainer (Falcon Franklin Lakes, NJ, USA). Erythrocytes were lysed with ACK lysing buffer (Invitrogen Corporation, Carlsbad, CA, USA) and cells were washed three times with DPBS. Liver peripheral blood cells were removed by left ventricle perfusion with PBS. After mechanical disruption of the liver, lymphocytes were isolated using 37.5% Percoll (GE Healthcare, Barrington, IL, USA) density centrifugation. Cells were washed with PBS and counted with trypan blue to exclude dead cells. Cells were flow cytometrically analyzed for cell membrane marker expression and for cytokine production. For the latter, cells were incubated at 37°C and 5% CO_2_ for 1 h in complete RPMI medium (this refers to RPMI1640 medium supplemented with 10% FCS, 100 U/ml penicillin, 100 µg/ml streptomycin, 2 mM glutamine and 50 µM 2-mercaptoethanol (all from Invitrogen Corporation)) supplemented with brefeldine A (1 µg/ml; Golgiplug, BD Biosciences). Cells were collected, cell-surface stained and subsequently permeabilized using Cytofix/Cytoperm reagent (BD Biosciences, San Jose, CA, USA) and intracellularly stained with anti-IFNγ and anti-TNFα mAb.

Spleen and brain were isolated from non-infected and from *P. berghei* ANKA-infected WT and Ly49E KO mice at the time of paralysis. Spleen isolation occurred as described above. The brain was isolated after left ventricle perfusion with PBS to remove peripheral blood from the brain. The brain was mechanically disrupted, minced and passed through a 70 µm filter. Brain lymphocytes were isolated by using 40%/70% Percoll density centrifugation. All cell suspensions were counted with trypan blue to exclude dead cells and were flow cytometrically analyzed.

### Antibodies

Monoclonal antibodies used for labeling were anti-NK1.1 (phycoerythrin-cyanine-7-conjugated, clone PK136), anti-CD49b (allophycocyanin (APC)-conjugated, clone DX5), anti-CD3 (pacific blue-conjugated, clone 145-2C11), anti-CD69 (PE-conjugated, clone H1.2F3), anti-γδ-TCR (fluorescein (FITC)-conjugated, clone GL3), anti-CD8α (APC-conjugated, clone Lyt-2), TNF-α (phycoerythrin (PE)-conjugated, clone MPG-XT22) and anti-IFN-γ (PE-conjugated, clone XMG1.2) (all from BD Biosciences, San Jose, CA, USA). Anti-Ly49E/C (biotin-conjugated, clone 4D12) [19], anti-Ly49E/F (FITC-conjugated, clone CM4, kindly provided by Dr. C.G. Brooks, Newcastle upon Tyne, U.K.) [18], anti-Ly49H (biotin-conjugated, clone 3D10, kindly provided by Dr. W. Yokoyama, St. Louis, MO, USA) and anti-Ly49A (FITC-conjugated, clone JR9-318, kindly provided by Dr. J. Roland, Paris, France). Anti-CXCR3 (PE-conjugated, clone 220803, R&D) and anti-CD4 (peridinin chlorophyll protein cyanine dye 5.5-conjugated, clone L3T4, Biolegend). Biotinylated mAbs were detected by streptavidin (APC-eFluor™780-conjugated, eBiosciences).

Before staining, the FcR was blocked with anti-FcγRII/III mAb (unconjugated, clone 2.4G2, kindly provided by Dr. J. Unkeless, New York, NY, USA). Live and dead cells were discriminated by using propidium iodide or the LIVE/DEAD® Fixable Aqua Dead Cell Stain Kit (Invitrogen Corporation) for analysis of cell membrane marker expression or cytokine production, respectively. Samples were measured using a BD LSR II flow cytometer and analyzed with FACSDiva 6.1.2 software (BD Biosciences).

### Statistical analysis

Data were statistically evaluated using PASW statistics 21 software (SPSS Inc., Chicago, IL, USA). Datasets were analyzed using the non-parametric 2-tailed Mann Whitney test or the Log-rank Kaplan-Meier method. A p-value<0.05 was considered statistically significant.

## Results

### Parasitemia of *Plasmodium berghei*-infected WT versus Ly49E KO mice

**Figure 1:**
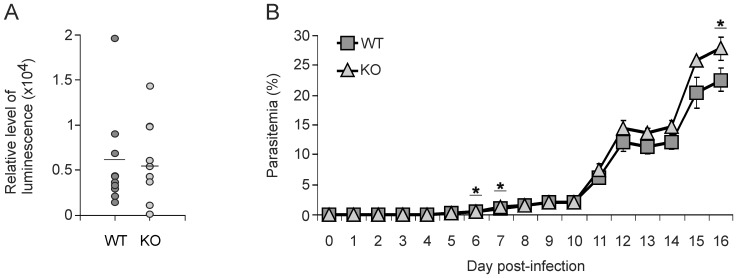
WT and Ly49E KO mice display a similar course of parasitemia. Mice were infected i.v. with 10,000 *P. berghei* ANKA sporozoites. (A) Liver parasitemia was determined at 44 h post-infection via luminescence imaging. Luminescence results are expressed as relative levels of luminescence. Each data point represents one individual mice; the horizontal bar represents the mean. The observed luminescence was 0.61×10^4^±0.17 and 0.54×10^4^±0.15 for WT and Ly49E KO, respectively (n = 10). These values were not significantly different using the 2-tailed Mann Whitney test. (B) Blood parasitemia was monitored daily by flow cytometric analysis of peripheral blood. The data presented were obtained by combined analysis of five experiments (WT, started with 89 mice and ended with 18; KO, started with 84 mice and ended with 16) and are shown as mean ± SEM. Statistical analysis was performed using the 2-tailed Mann Whitney test. * indicates a significant difference with p<0.05.

Using *P. berghei* ANKA sporozoites, which expresses a fusion protein of green fluorescent protein (GFP) and firefly luciferase, we determined parasitemia both in liver and in blood and compared WT to Ly49E KO mice. Based on a paper of Ploemen *et al*. [26] in which it is shown that the highest parasite load in the liver is observed at 44 h post-infection, we measured at this point in time the luminescence of the liver region via whole body imaging. The observed luminescence was 0.61×10^4^±0.17 and 0.54×10^4^±0.15 for WT and Ly49E KO, respectively. These values were not significantly different (Figure 1A). In addition, we determined blood parasitemia levels in WT and Ly49E KO mice by flow cytometric analysis of the percentage of GFP-positive RBCs. Parasitemia was detectable from day 5 p.i., after which only a minor increase was observed until day 10 p.i. (Figure 1B). Subsequently, parasitemia increased drastically and peaked at days 12 and 16 p.i., confirming the results of Hansen *et al* [27]. There was a small, but significantly increased percentage of *P. berghei* ANKA-infected RBCs in Ly49E KO as compared to WT mice at days 6 (0.49%±0.042 vs 0.40%±0.041) and 7 p.i. (1.20%±0.072 vs 1.07%±0.074). However, as these differences are very small, it can be questioned whether they are functionally relevant. This difference was also no longer observed from day 8 p.i., with the exception of day 16 p.i. where again a higher parasitemia was observed in Ly49E KO mice (27.75%±1.89 vs WT: 22.57%±1.85).

### Phenotypical and functional analysis of liver and spleen lymphocytes of WT versus Ly49E KO mice at the early phase of *P. berghei* ANKA infection

**Figure 2:**
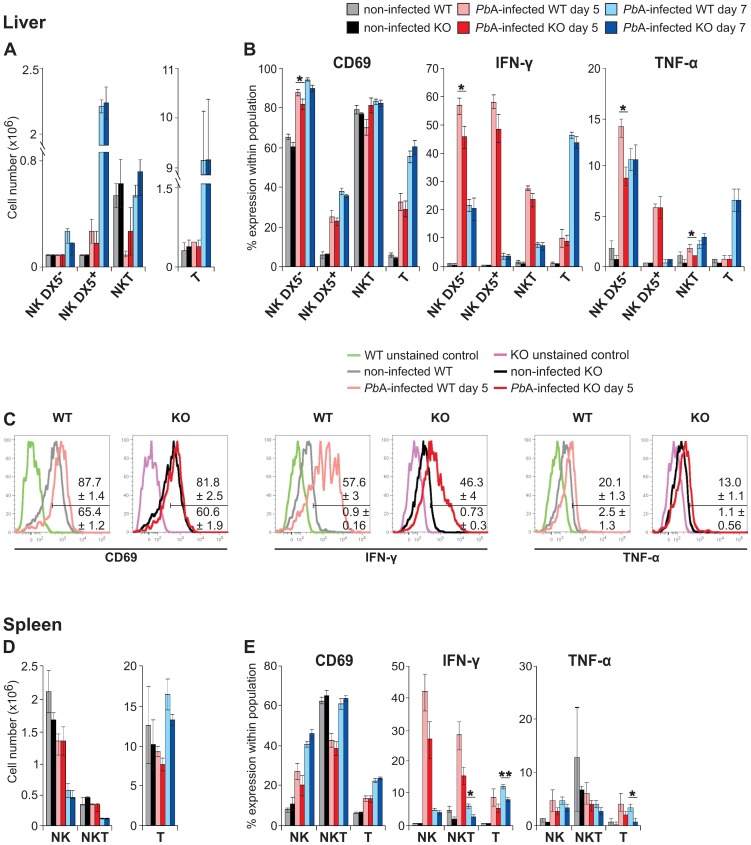
Role of Ly49E expression in lymphocyte phenotype and cytokine production in early *P. berghei* ANKA infection. Liver lymphocytes (A–C) and spleen cells (D–E) were isolated from non-infected, or from 5 d and 7 d *P. berghei* ANKA-infected WT and Ly49E KO mice, as indicated in the figure. Freshly isolated liver lymphocytes (A) and spleen cells (D) were analyzed for their subpopulation composition by gating on NK cells (liver: CD3^−^NK1.1^+^DX5^−^ and CD3^−^NK1.1^+^DX5^+^; spleen: CD3^−^NK1.1^+^), NKT cells (CD3^dim^NK1.1^+^) and T cells (CD3^+^NK1.1^−^). The absolute cell number was calculated based on the total viable cell number obtained after isolation and the representative percentages of each cell population. CD69, IFN-γ and TNF-α expression by liver (B) and spleen (E) NK, NKT and T cells was determined. (A,B,D,E) Data are represented as mean ± SEM (n = 5). Statistical analysis was performed using the 2-tailed Mann Whitney test. * indicates a significant difference with p<0.05 and ** a significant difference with p<0.01. (C) Overlay histograms show unstained, and CD69-, IFN-γ- and TNF-α-stained DX5^−^ liver NK cells from non-infected and day 5 *P. berghei* ANKA-infected WT and Ly49E KO mice, as indicated. The histograms of one representative mouse of each group is shown. The mean ± SEM of 5 different mice is shown for *P. berghei* ANKA-infected (above the gate bar) and for non-infected mice (below the gate bar).

Roland *et al*. [4] showed that at days 5 and 7 p.i. the liver NK cell number increases, while the spleen NK cell number decreases. As Takeda *et al*. [28] have shown that liver NK cells can be subdivided on the basis of DX5 expression, and as we have shown that Ly49E is preferentially expressed by DX5^−^ liver NK cells [25], we compared the absolute number of DX5^−^ and DX5^+^ liver NK cells in *P. berghei* ANKA-infected WT vs Ly49E KO mice at days 5 and 7 p.i. (Figure 2A). As also liver T and NKT cells are involved in the immune response towards *P. berghei* ANKA infection [29]; [30], these were analyzed in parallel. As expected, we observed an increase in the liver NK cell number at day 5 p.i. and, more pronounced, at day 7 p.i. The strong increase of the liver NK cell number at day 7 p.i. was mainly due to an increase of the DX5^+^ subpopulation. There was no significant difference between WT and Ly49E KO mice. Whereas no change in the liver NKT cell number was observed, there was a strong increase in the liver T cell number at day 7 p.i., which was similar for WT vs Ly49E KO mice (Figure 2A). In the spleen, we observed a decrease in the NK cell number in *P. berghei* ANKA-infected mice, confirming the results of Roland *et al*. [4], and which was comparable in WT vs Ly49E KO. No significant difference in spleen NKT and T cell numbers was observed between WT and Ly49E KO mice (Figure 2D).

Next we investigated the activation status of hepatic and splenic lymphocytes. Expression of the early activation marker CD69 was clearly increased on both DX5^−^ and DX5^+^ liver NK cells, spleen NK cells and liver and spleen T cells of *P. berghei* ANKA-infected mice at days 5 and 7 p.i. (Figure 2B;E). IFN-γ production was highly induced in both DX5^−^ and DX5^+^ liver NK cells, spleen NK cells, and in liver and spleen NKT and T cells of *P. berghei* ANKA-infected mice. TNF-α was mainly produced by liver NK cells. The comparison of WT and Ly49E KO mice shows lower expression of CD69, IFN-γ and TNF-α in DX5^−^ liver NK cells of Ly49E KO mice at day 5 p.i. (Figure 2B–C). For splenic T cells, there was a significant decrease in IFN-γ and TNF-α production in infected Ly49E KO mice compared to WT mice at day 7 p.i. (Figure 2E).

**Figure 3:**
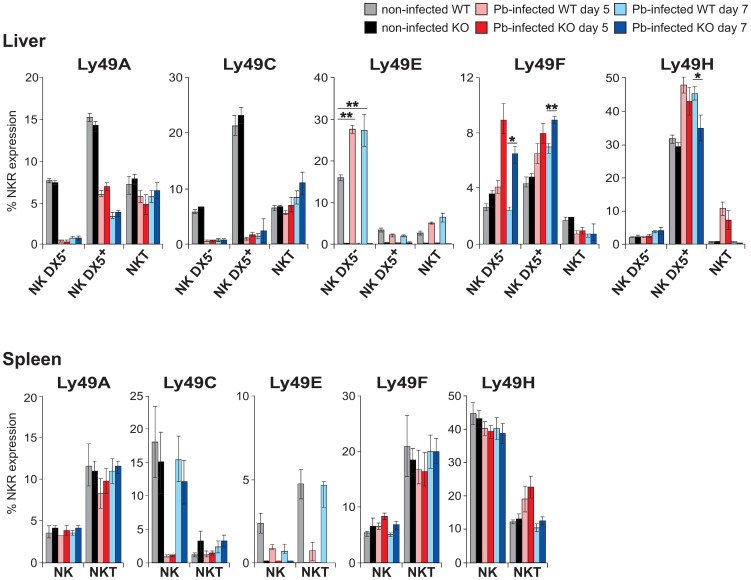
NK receptor expression on NK and NKT cells in early *P. berghei* ANKA infection. NK cell receptor expression is shown for the indicated gated lymphocyte subpopulations of liver and spleen. The expression of Ly49E was determined by gating on Ly49E/C (mAb 4D12) and Ly49E/F (mAb CM4) double-positive cells. Data are represented as mean percentage ± SEM (n = 5). Statistical analysis was performed using the 2-tailed Mann Whitney test. * indicates a significant difference with p<0.05 and ** a significant difference with p<0.01.

In WT mice, Ly49E expression was clearly upregulated upon infection in liver DX5^−^ NK cells and NKT cells at day 5 p.i., and remained high at day 7 p.i. (Figure 3). In addition to Ly49E, NK and NKT cells also express other members of the Ly49 family, of which we analyzed the inhibitory Ly49A, Ly49C and Ly49F and the activating Ly49H receptors. Compared to WT mice, a significantly higher percentage of Ly49E KO DX5^−^ and DX5^+^ liver NK cells expressed Ly49F, whereas a significantly lower percentage of Ly49E KO DX5^+^ liver NK cells expressed Ly49H at day 7 p.i. There was no significant difference in expression of other NK receptors on spleen NK, NKT and T cells from WT vs Ly49E KO mice upon *P. berghei* ANKA infection (Figure 3).

### Development of cerebral malaria in WT versus Ly49E KO mice

**Figure 4:**
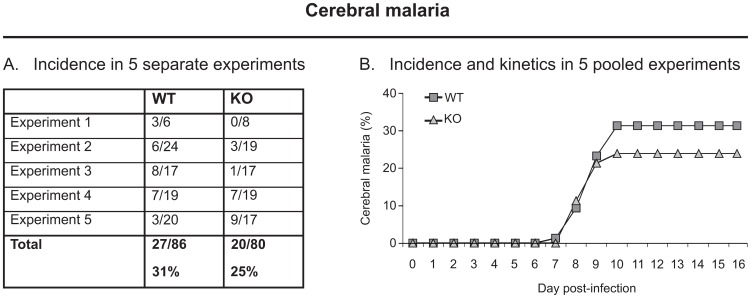
Cerebral malaria development in *P. berghei* ANKA-infected WT vs Ly49E KO mice. *P. berghei* ANKA-infected WT and Ly49E KO mice were monitored daily for cerebral malaria development. (A) The incidence of cerebral malaria development in 5 separate experiments. The results are presented as (number of animals with cerebral malaria)/(total number of animals studied). The last line represents the total number of mice affected with cerebral malaria during these 5 experiments. (B) The cumulative incidence and kinetics of cerebral malaria development in the 5 pooled experiments (WT, n = 86; Ly49E KO, n = 80). Cerebral malaria development results were analyzed by Log-rank (Kaplan-Meier). There was no significant difference in WT vs Ly49E KO mice.

Upon i.v. infection with 10,000 *P. berghei* ANKA sporozoites of both WT and Ly49E KO mice, mice were monitored daily and evaluated for neurological signs such as loss of reflex, ataxia and paralysis, reflecting the development of cerebral malaria. Development of cerebral malaria was studied in five separate experiments. The percentage of animals developing cerebral malaria in each individual experiment is shown in Figure 4A, and the kinetics of cerebral malaria development of the 5 pooled experiments, consisting of 86 WT and 80 Ly49E KO mice, are presented in Figure 4B. The first animals developing cerebral malaria were observed at day 7 and day 8 p.i. in WT and Ly49E KO mice, respectively, whereas the last animals that developed cerebral malaria were observed at day 10 p.i. in both groups of mice (Figure 4B). No significant difference was observed in the kinetics and the percentage of cerebral malaria development in WT vs Ly49E KO mice (p = 0.325, Kaplan-Meier Log-rank method). The observed incidence of cerebral malaria upon infection with sporozoites was around 30%, whereas others [31]–[34] have reported an incidence of cerebral malaria of approximately 90% upon injection of infected RBCs. We also tested this in the Ly49E KO and WT mice and observed an incidence of cerebral malaria of 80–90%, with no difference between WT and Ly49E KO mice (data not shown).

### Phenotypical and functional analysis of spleen and brain lymphocytes of mice with cerebral malaria

**Figure 5:**
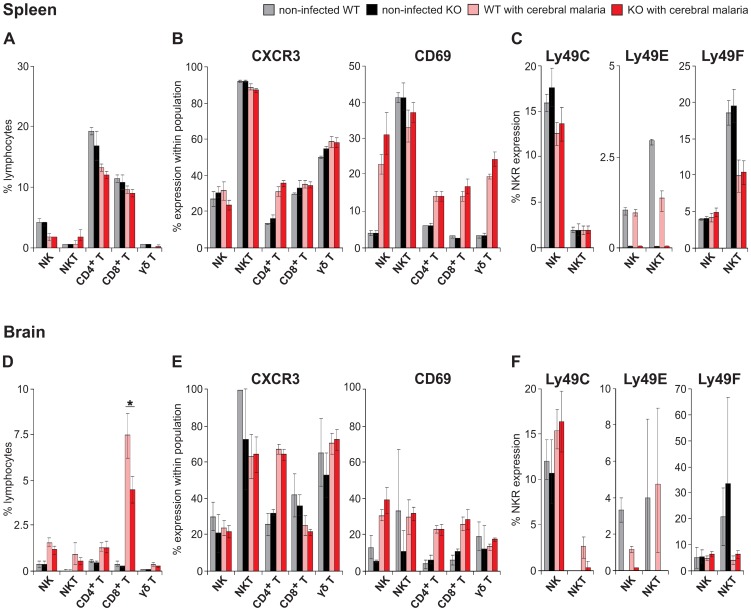
Analysis of spleen and brain lymphocytes of cerebral malaria developing *P. berghei* ANKA-infected WT vs Ly49E KO mice. Freshly isolated spleen (A–C) or brain (D–F) NK (CD3^−^NK1.1^+^), NKT (CD3^dim^NK1.1^+^), CD4^+^ T (CD3^+^CD4^+^), CD8^+^ T (CD3^+^CD8^+^) and γδ T cells (CD3^+^TCRγδ^+^) from non-infected WT and Ly49E KO mice or cerebral malaria developing *P. berghei* ANKA-infected WT and Ly49E KO mice (as indicated in the figure) were analyzed by flow cytometry. The percentage of these lymphocyte populations are shown in A and D. CXCR3 and CD69 expression was analyzed within each lymphocyte population (B and E). NK receptor expression was determined in NK and NKT cells (C and F). Data represent the results of 2 independent experiments (WT, n = 8; KO, n = 11) and are shown as mean ± SEM. Statistical analysis was performed using the 2-tailed Mann Whitney test. * represent a significant difference with p<0.05.

It has been shown that mice that develop cerebral malaria have a decrease in splenic NK cells, and an increase in brain NK cells [7]. Also CD4^+^ and in particular CD8^+^ T cells migrate to the brain. The chemokine receptor CXCR3, which is crucial for this migration, is upregulated on αβ-T cells in an NK cell-dependent manner [7]. In our study, we analyzed by flow cytometry spleen and brain lymphocytes from WT and Ly49E KO mice at the day of paralysis, and compared them to non-infected mice. In agreement to Hansen *et al*. [7], we showed that during cerebral malaria splenic NK cells decreased, while the percentage of brain NK cells increased (Figure 5A+D). Also γδ T, CD4^+^ T and, more pronounced, CD8^+^ T cells were significantly increased in the brain of mice that developed cerebral malaria compared to non-infected mice (Figure 5D). Significantly less CD8^+^ T cells accumulated in the brain of Ly49E KO mice with cerebral malaria compared to affected WT mice (Figure 5D).

As CXCR3 is a crucial chemokine receptor in the migration of T cells to the brain during cerebral malaria, we studied its expression in spleen and brain lymphocytes. CXCR3 expression was increased on spleen and brain CD4^+^ T cells in cerebral malaria mice compared to non-infected mice (Figure 5B;E). There were no significant differences between WT and Ly49E KO mice. We also analyzed CD69 expression. As expected, CD69 expression was increased on spleen and brain NK and T cell subpopulations. No significant difference was observed between WT and Ly49E KO mice (Figure 5B;E). Finally, NK receptor expression analysis on NK and NKT cells shows that there were no differences in cerebral malaria vs non-infected mice (Figure 5C;F).

## Discussion

This study shows that deficiency in Ly49E expression results in lower parasitemia at the early stage of *P. berghei* ANKA infection, i.e. at days 6–7 p.i., which correlates with lower IFN-γ and TNF-α expression in liver NK cells. These differences are, however, lost thereafter. Also cerebral malaria develops similarly in WT vs Ly49E KO mice. Collectively, we show that Ly49E, which is a regulatory NK receptor expressed on liver NK and NKT cells, only has a temporarily role in the host immune response against *P. berghei* ANKA infection.

It has been shown that liver NK cells are involved in the early immune response controlling *Plasmodium* infection. Comparison of the outcome of *Plasmodium* infection in RAG2^−/−^ mice, which have normal NK cell numbers but lack T and B cells, and RAG2^−/−^ γc^−/−^ mice, which lack T, B and NK cells, showed that NK cells act on the pre-erythrocytic phase of parasite development *in vivo* as RAG2^−/−^ γc^−/−^ mice are more sensitive to sporozoite infection than RAG2^−/−^ mice, whereas survival rates are similar for the two strains following infection with parasitized RBCs [4]. The role of NK cells in the later stage of infection, and in cerebral development in particular, has been previously evaluated with somewhat contradictory results. Hansen *et al*. [7] showed that depletion of NK cells by anti-asialo GM1 antibody protects animals against *P. berghei* ANKA-mediated cerebral malaria, while parasitemia is increased in these animals. On the contrary, Yanez *et al*. [32] reported that depletion of NK cells with anti-NK1.1 antibody fails to protect mice from *P. berghei* ANKA-mediated cerebral malaria. However, usage of anti-NK1.1 antibody results in crosslinking of the NK1.1 receptor and in IFN-γ production [35]. Whereas IFN-γ is protective in the early phase of infection [6]; [36]; [37], it provokes enhancing effects on cerebral malaria development [31]. Also NKT cells, which coexpress NK receptors and T cell receptors, have been implicated in the regulation of cerebral malaria. CD1^−/−^ or Jα281^−/−^ C57BL/6 mice, which lack CD1-restricted and Vα14 NKT cells, respectively, are partially protected from cerebral malaria development upon injection of *P. berghei* ANKA-parasitized RBC. This correlates with decreased IFNγ production as compared to WT mice [38].

All these data indicate a substantial role for NK and NKT cells in the control of *P. berghei* ANKA infection, either directly via secretion of pro-inflammatory effector cytokines or indirectly by inducing T_H_1 polarization. The activity of NK cells, and also partially of NKT cells, is regulated by NK receptors, which can either be activating or inhibitory. In mice, NK receptors are encoded by a genetic locus, i.e. the NK complex, which is located on chromosome 6. Among other genes, the NK complex is comprised of the *Cd94* gene and of the multigene families *Nkg2* and *Ly49*. The balance between the signaling of activating and inhibitory CD94/NKG2 and Ly49 receptors determines whether NK cell activation will occur. Ly49E is a unique member of the Ly49 receptor family, with several distinctive characteristics. Unlike other Ly49 receptors, Ly49E does not bind classical MHC class-I ligands [16]. Instead, Ly49E is triggered through interaction with the non-MHC class-I molecule uPA [17]. Another unique characteristic is that DX5^−^ liver NK cells express high levels of Ly49E, whereas other Ly49 members are virtually absent [25]. Collectively, these characteristics pointed towards a possible regulatory role for Ly49E in malaria pathogenesis. Also supporting this hypothesis was that uPA contributes to the mortality in *P. berghei* ANKA infected mice. Piguet *et al*. [33] argues that this is due to increased platelet trapping and thus thrombocytopenia. We hypothesized an additional explanation as uPA, which we showed to trigger the Ly49E receptor [17], thus also exerts its effects by regulation of Ly49E-expressing immune cells.

Whereas Ly49E is highly expressed on DX5^−^ liver NK cells, its expression on NK cells in peripheral blood, spleen and lymph nodes is very low in adult mice [18]; [19]; [25]. However, Ly49E expression can be induced on spleen NK cells from adult mice after *in vitro* culture in the presence of IL-2 or IL-15 [39]. Given this differential expression of Ly49E in liver versus other peripheral NK cells, we expected to observe Ly49E-mediated immune effects mainly during the early stage of *Plasmodium* infection, which starts with the liver stage. We therefore injected the mice with *P. berghei* ANKA sporozoites instead of *P. berghei* ANKA-parasized RBCs, as injected sporozoites start the infection with the liver stage, whereas with parasized RBCs the liver stage is skipped and infection directly starts from the blood stage. As expected [4]; [7], we observed an increase in the liver NK cell number at day 5 p.i. and, more pronounced, at day 7 p.i. There was also a strong increase in the liver T cell number at day 7 p.i. This increase in liver NK and T cell numbers was comparable in WT vs Ly49E KO mice. Importantly, in WT mice, Ly49E expression was clearly upregulated in liver DX5^−^ NK cells and NKT cells at day 5 p.i., and remained high at day 7 p.i. We observed significantly increased parasitemia in Ly49E KO as compared to WT mice at days 6 and 7 p.i. This correlated with lower expression of the protective cytokines IFN-γ and TNF-α in DX5^−^ liver NK cells of Ly49E KO mice at day 5 p.i. However, these differences were not observed in the later stage of infection. Also cerebral pathology, in which leukocyte and platelet sequestration in the brain blood vessels occurs and for which a causal role has been demonstrated for the cytokine IFNγ [31] and for NK, NKT and T cells [7]; [32]; [38], developed with similar kinetics and incidence in WT vs Ly49E KO mice. Cumulatively, our findings point towards a regulating role for the Ly49E receptor in the early host response towards *P. berghei* ANKA sporozoite infection but, although significant, it is a relatively minor effect which fades during the later stage of infection.

*P. berghei* ANKA infection results in cerebral malaria depending on the genetic background of the mice as C57BL/6 mice are highly susceptible, while BALB/c mice are resistant. In a very elegant study, Hansen *et al*. [27] have shown that the NK receptor complex determines susceptibility to *P. berghei* ANKA-mediated cerebral malaria as congenic BALB.B6-Cmv1^r^ mice, in which the NK complex from C57BL/6 mice has been introduced in the BALB/c background, have an increased rate of cerebral malaria development as compared to resistant BALB/c mice. The NK complex regulates murine cerebral malaria by a mechanism independent of parasite growth rates, as parasitemia levels are unchanged. The NK complex spans a region of approximately 4.7 mB in which several genes are present, including the multigene families *Nkg2* and *Ly49*. Although Ly49E is a member of the Ly49 family, it was unlikely that Ly49E is responsible for the observed increased cerebral malaria incidence in congenic BALB.B6-Cmv1^r^ mice as, in contrast to the extensive allelic polymorphism of other *Ly49* genes, *Ly49e* is highly conserved between mouse strains, and also has an identical sequence in C57BL/6 and BALB/c mice [40]. In our study, in which the C57BL/6 background was used, Ly49E KO mice developed cerebral malaria to the same extent and with similar kinetics as WT mice. This shows that another gene, or a set of genes, in the NK complex is responsible for the cerebral malaria susceptibility.

In conclusion, although there are several indications that the Ly49E receptor could play a crucial regulatory role in the host immune response towards *P. berghei* ANKA infection, we show that Ly49E-deficient mice only display a temporarily increased parasitemia, which correlates with decreased activation of liver NK cells and cytokine production. The subsequent stage of infection, including cerebral malaria development, is not affected. From these data we conclude that Ly49E expression only has a temporarily role in the immune control of *Plasmodium* pathogenesis.

## Acknowledgments

We would like to thank Dr. Robert Sauerwein for providing us with *P. berghei* ANKA-infected mosquitoes. We also thank Jet Robin and Eelke Vandenberghe for invaluable help with animal housing and care.
